# Supplementary material for: Host genetics and diet jointly shape the microbiome of Drosophila melanogaster but do not predict lifespan or age-related traits
Source: Biogerontology. 2026 May 4;27(3):99. doi: 10.1007/s10522-026-10431-2 (PMC13139259; doi:10.1007/s10522-026-10431-2)
Supplement: Supplementary file 1 — Supplementary file1 (DOCX 4228 KB) [file 10522_2026_10431_MOESM1_ESM.docx]

Supplementary material for

**Host genetics and diet jointly shape the microbiome of *Drosophila melanogaster* but do not predict lifespan or age-related traits**

­

This supplementary file contains:

Supplementary Figures

Figure S1 Genera relative abundance for lines on control and restricted diet

Figure S2 Species relative abundance for lines on control and restricted diet

Figure S3 Correlation plots between diets for genera and species

Figure S4 Thredshold for sample cutoff

Figure S5 Receiver Operating Characteristic (ROC) curves comparing *Wolbachia* sequence read counts to expected infection status across dietary treatments

Figure S6 Comparison of distance measures used in CCA

Figure S7 PCA distance measure comparison for flies on a control diet

Figure S8 PCA distance measure comparison for flies on a restricted diet

Supplementary Tables

Table S1 Summary of ANOVA on data of unique OTU

Table S2 Summary of ANOVA on data of Simpson Index

Table S3 Summary of ANOVA on data of Shannon Index

Table S4 Summary of ANOVA on data of relative abundance of genera [Excel]

Table S5 Summary of ANOVA on data of relative abundance of species [Excel]

Table S6 Correlation between unique OTUs, Simpson Index and Shannon Index with age-related traits [Excel]

Table S7 Correlation between relative abundance of genera and age-related traits [Excel]

Table S8 Correlation between relative abundance of species and age-related traits [Excel]

Table S9 Correlation between PC1 and PC2 scores with age-related traits [Excel]
 Table S10 Genotype number of the DGRP lines obtained from the Bloomington *Drosophila* Stock Center [Excel]


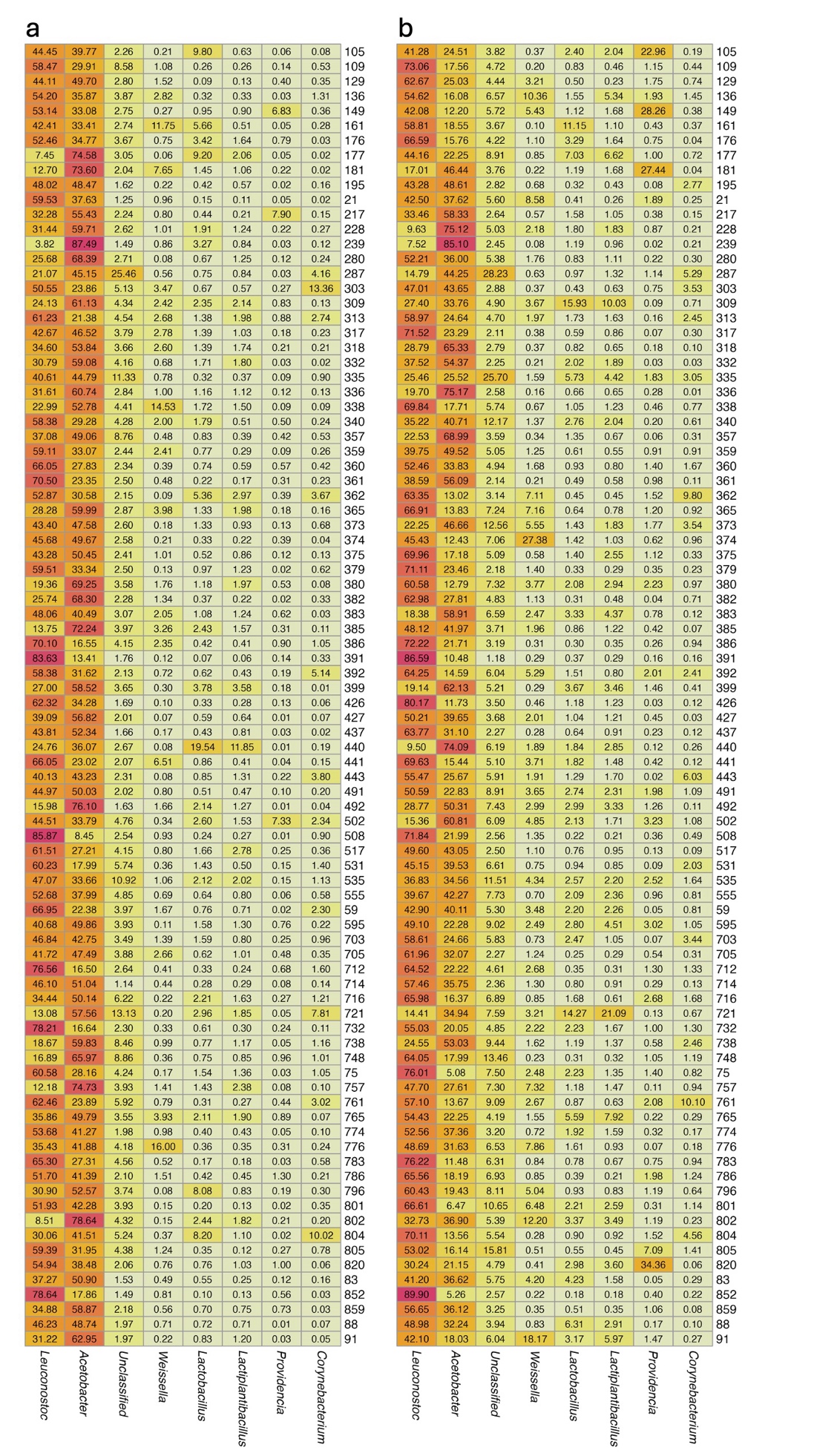


**Fig. S1 Genera relative abundance for lines on control and restricted diet.** The heatmaps shows genera for the separate DGRP lines on (**a**) the control diet and (**b**) the restricted diet. Genera was kept if they exhibited at least 1% relative abundance in flies from at least one of the two dietary conditions. Relative abundance is represented as a percentage, with dark red indicating the highest abundance, yellow indicating medium abundance, and olive green indicating the lowest abundance


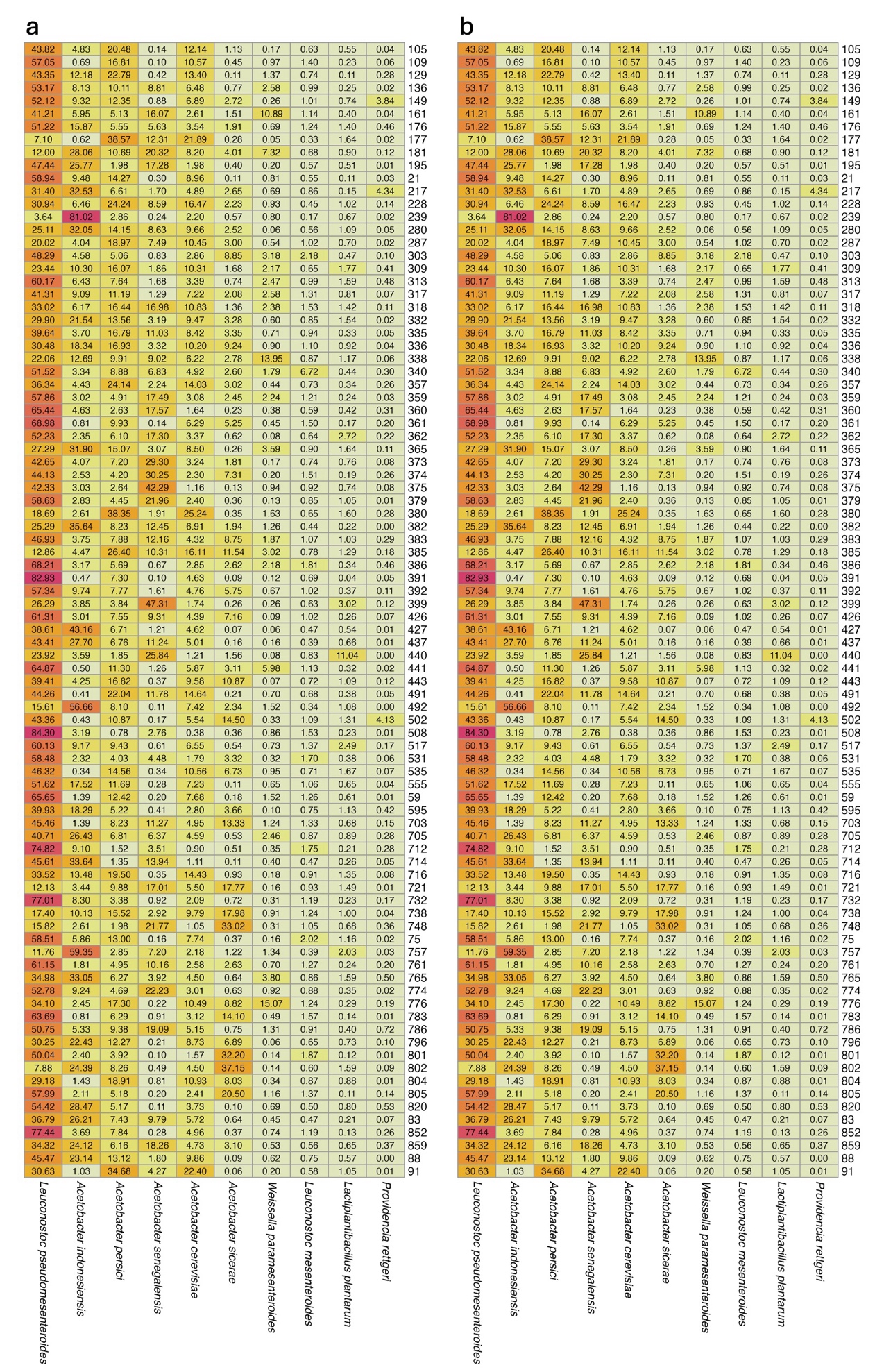


**Fig. S2 Species relative abundance for lines on control and restricted diet.** The heatmaps shows species for the separate DGRP lines on (**a**) the control diet and (**b**) the restricted diet. Species was kept if they exhibited at least 1% relative abundance in flies from at least one of the two dietary conditions. Relative abundance is represented as a percentage, with dark red indicating the highest abundance, yellow indicating medium abundance, and olive green indicating the lowest abundance


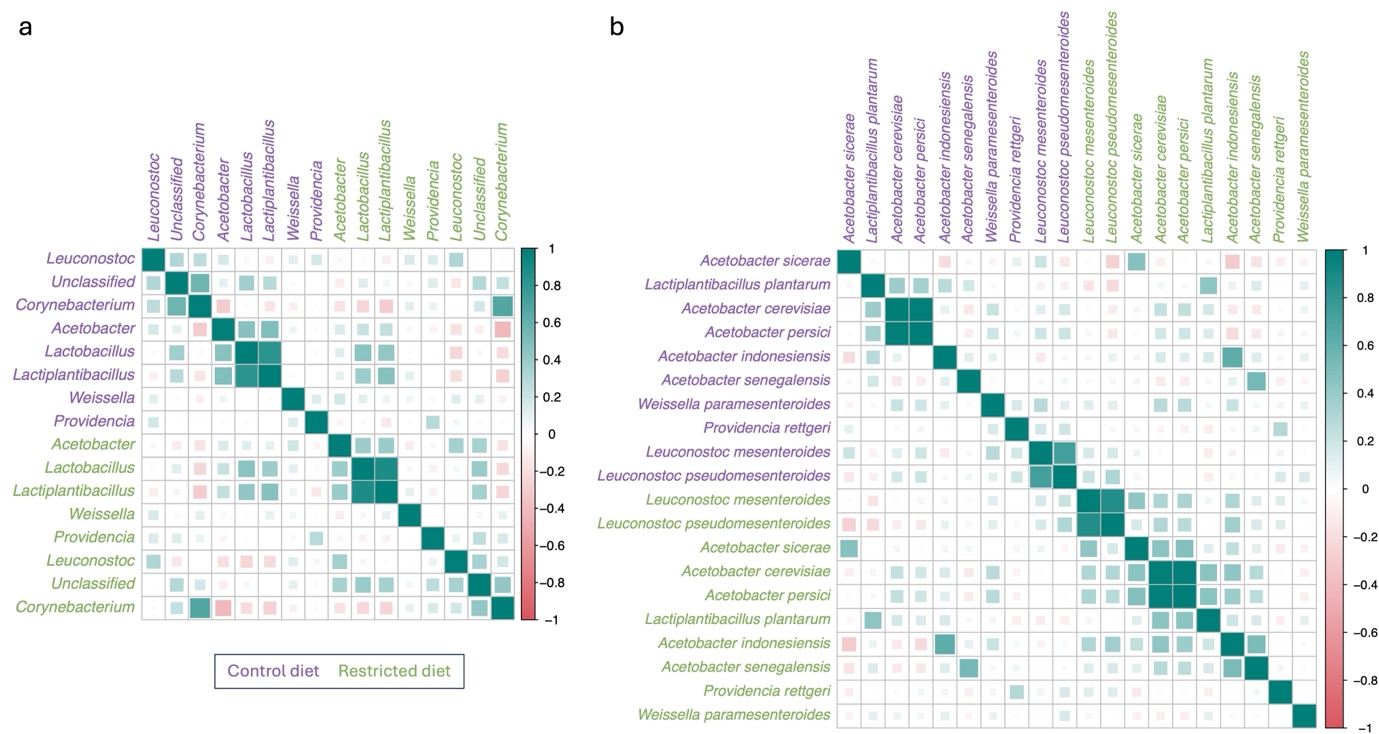


**Fig. S3 Correlation plots between diets of genera and species.** Spearman correlations coefficient for the relative abundance of (**a**) genera and (**b**) species, with relative abundances above 1%, between control diet (purple) and restricted diet (green). The color and size of the markers represent the strength and direction of the correlations, green indicates positive correlations, red indicates negative ones, and larger markers denote stronger correlations

**
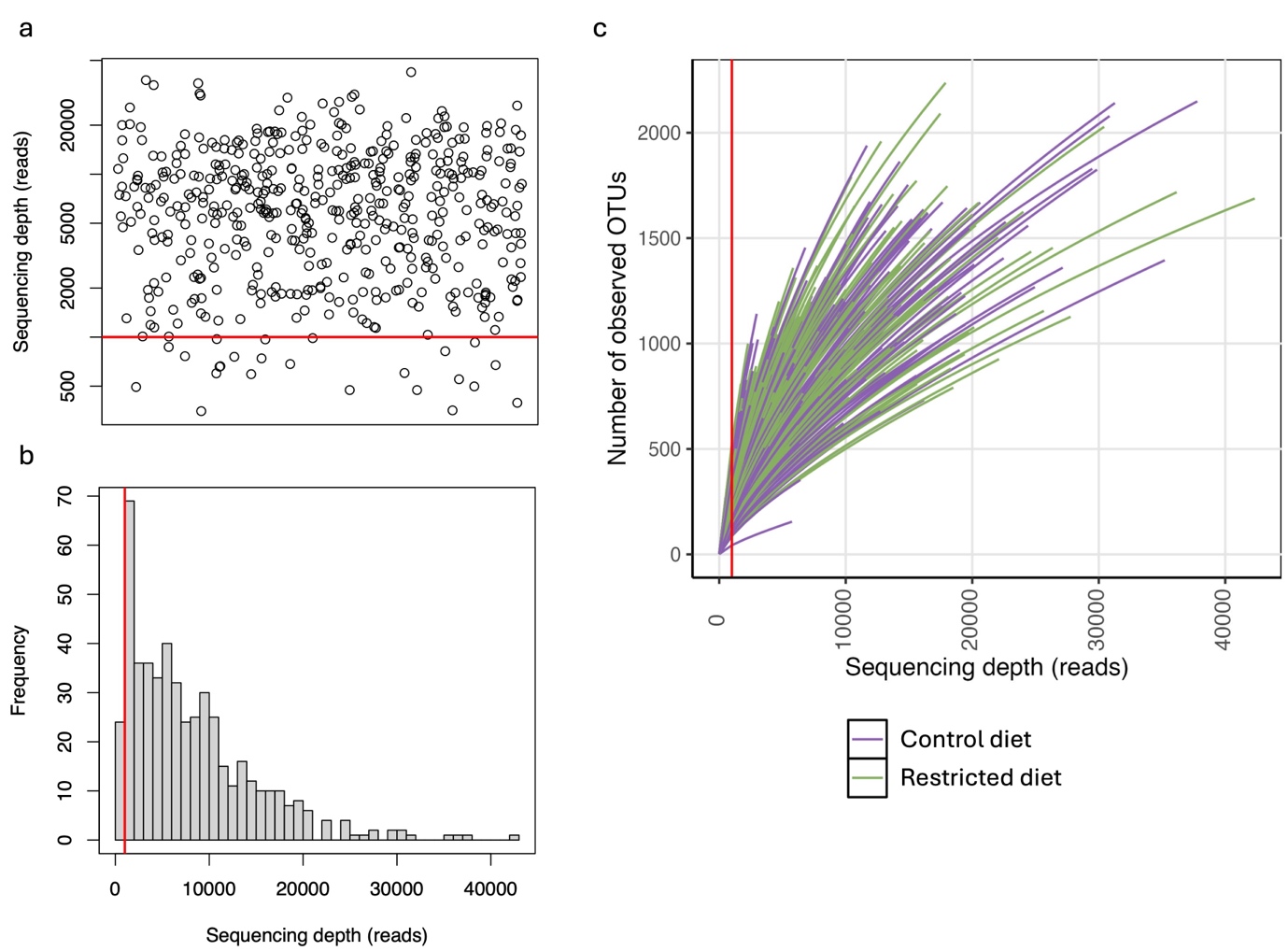
Fig. S4 Threshold for sample cutoff.** Sequencing depth was visualised using (**a**) a jitter plot, (**b**) a histogram and (**c**) a rarefaction curve for flies maintained on the control diet (purple) and on the restricted diet (green). Red line indicates a sequencing depth of 1000 reads


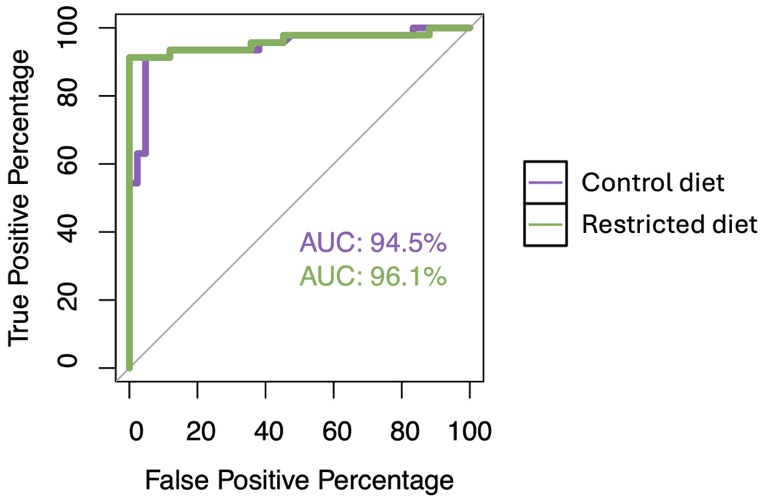


**Fig. S5 Receiver Operating Characteristic (ROC) curves comparing *Wolbachia* sequence read counts to expected infection status across dietary treatments.** The ROC curve for flies maintained on the control diet (purple) yield an Area Under the Curve (AUC) of 94.5%, while the curve for flies on the restricted diet (green) with an AUC of 96.1%


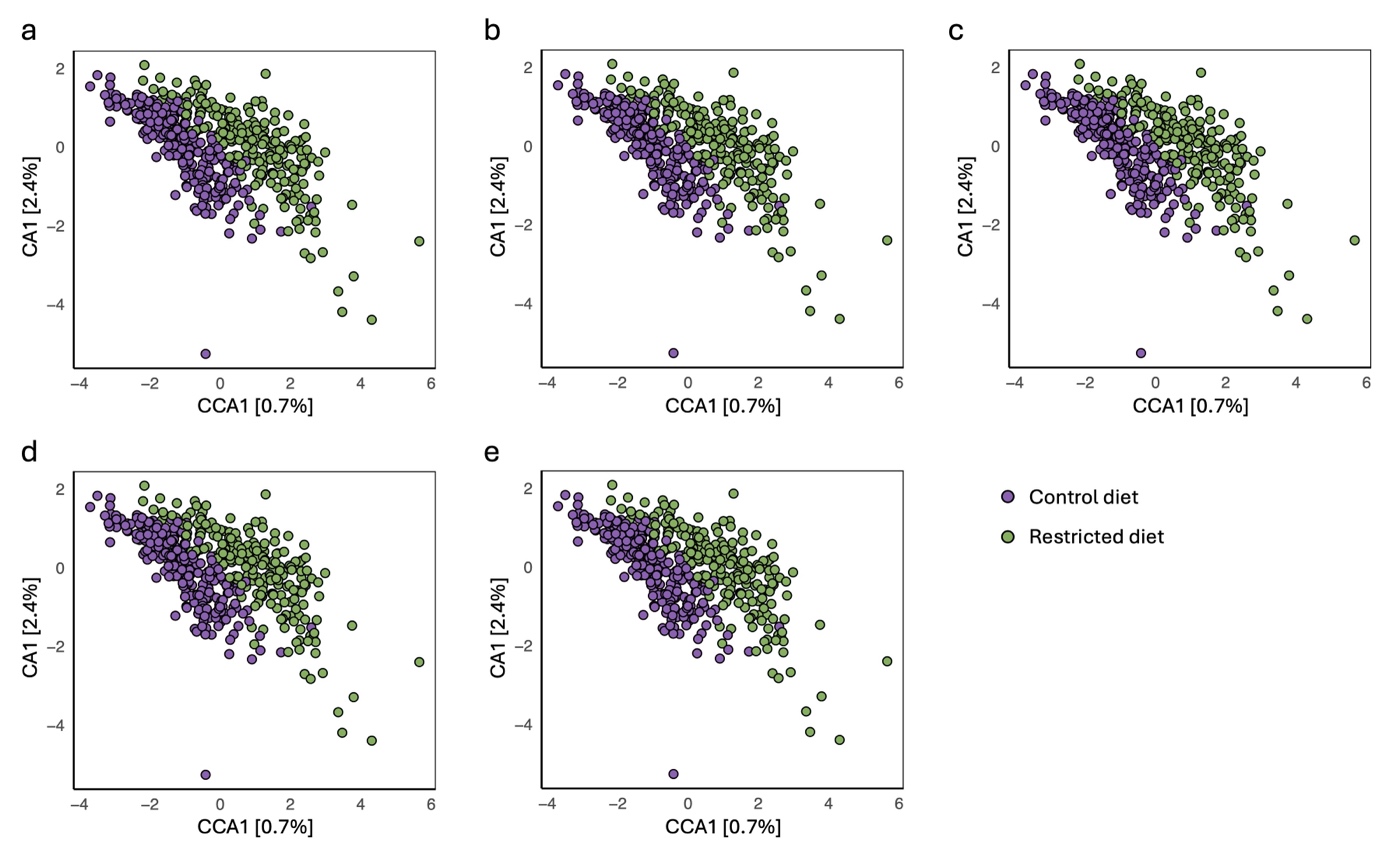


**Fig. S6 Comparison of distance measures used in CCA/CA plot.** Panels show CCA/CA plots based on five different distance metrics: (**a**) Aitchison, (**b**) Chi-square, (**c**) Chord, (**d**) Euclidean, and (**e**) Robust Aitchison. Each plot displays the first canonical axis (CCA1) on the x-axis and the first correspondence axis (CA1) on the y-axis, with the percentage of variance explained indicated in brackets. Data points are color-coded by diet group: control (purple) and restricted (green)


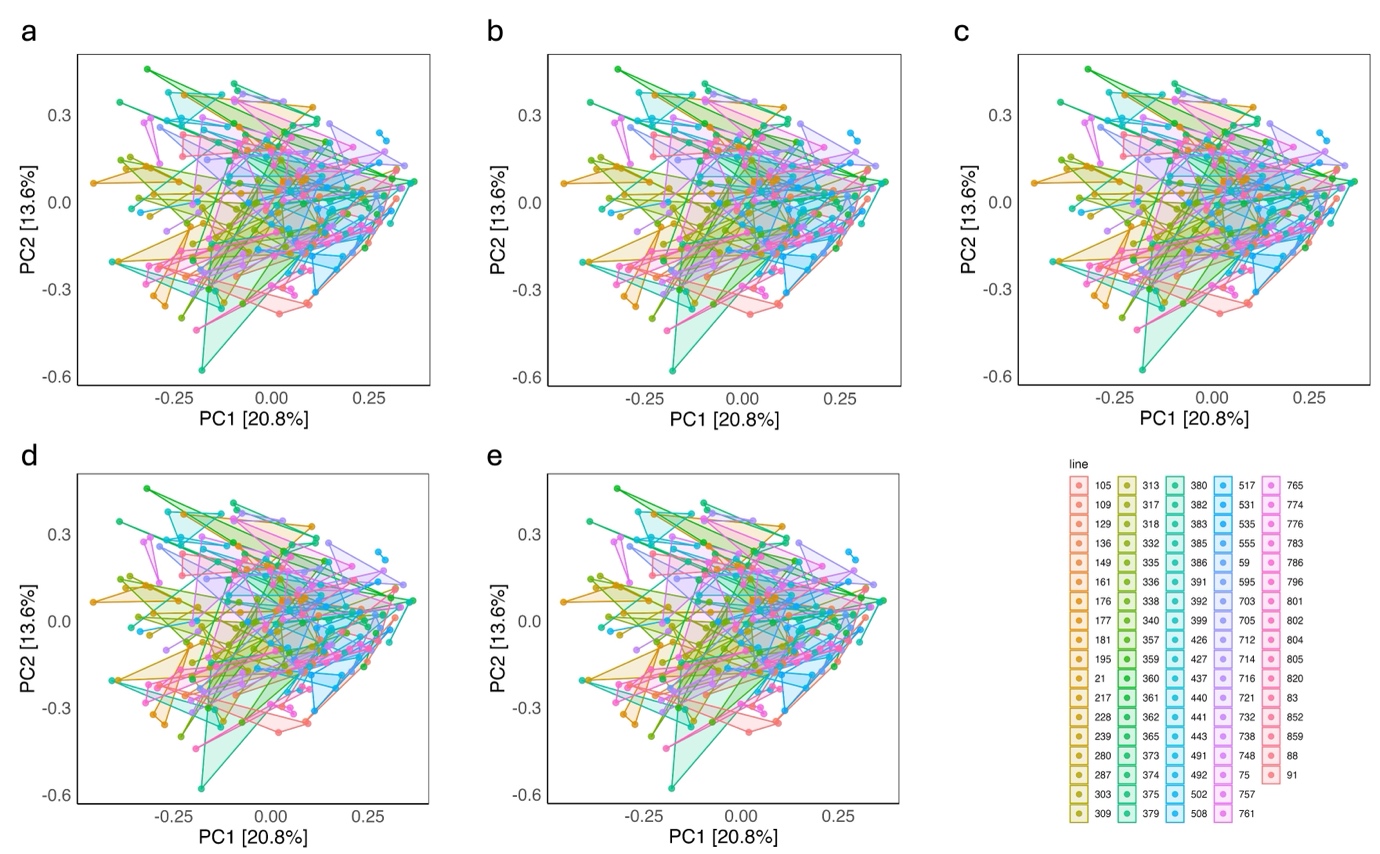


**Fig. S7 PCA distance measure comparison for flies on a control diet.** PCA plots are shown for five different distance metrics: (**a**) Aitchison, (**b**) Chi-square, (**c**) Chord, (**d**) Euclidean, and (**e**) Robust Aitchison. Each plot displays the first two principal components (PC1 and PC2), with the percentage of variance explained indicated on each axis. The colour gradient represents DGRP lines


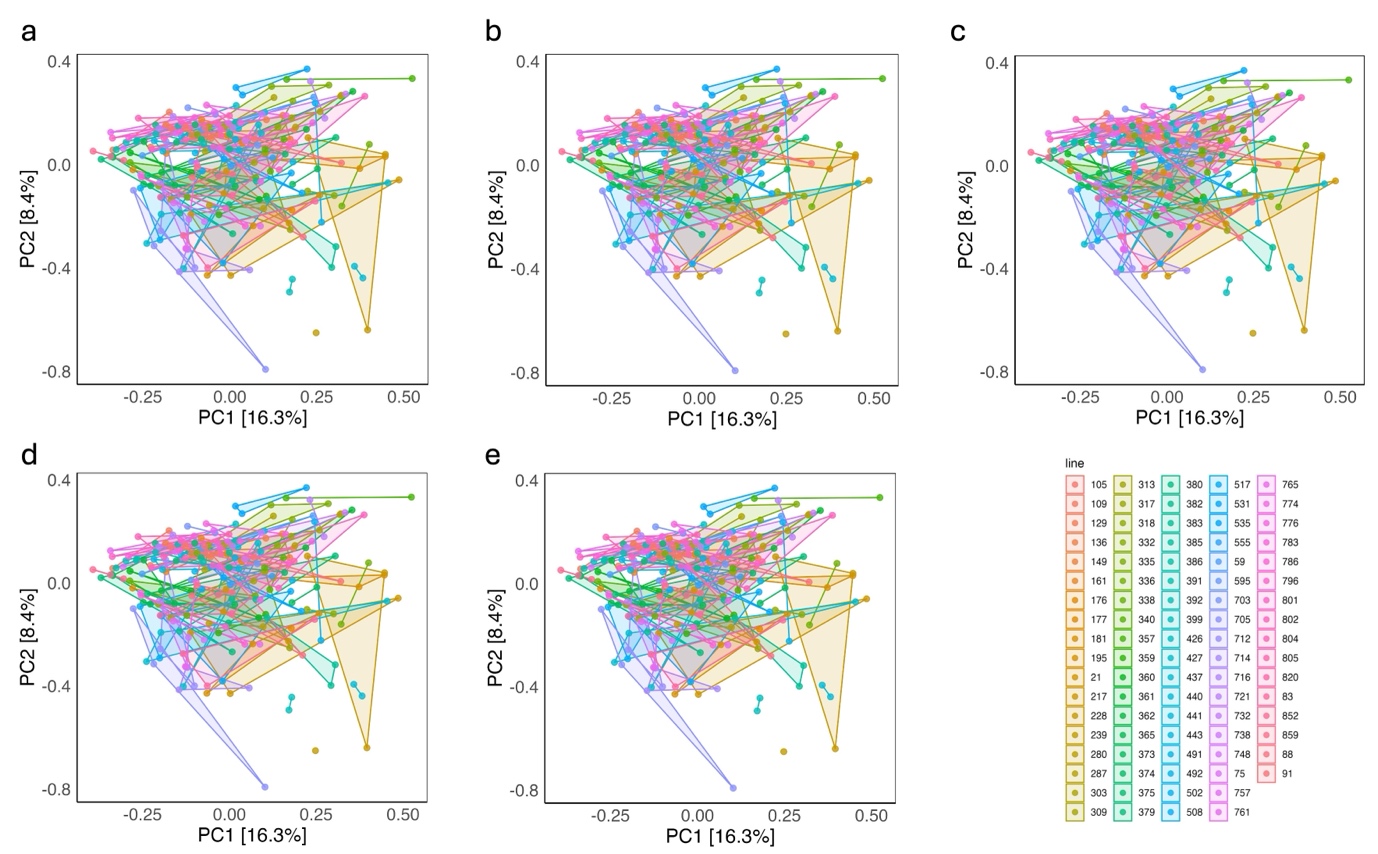


**Fig. S8 PCA distance measure comparison for flies on a restricted diet.** PCA plots are shown for five different distance metrics: (**a**) Aitchison, (**b**) Chi-square, (**c**) Chord, (**d**) Euclidean, and (**e**) Robust Aitchison. Each plot displays the first two principal components (PC1 and PC2), with the percentage of variance explained indicated on each axis. The colour gradient represents DGRP lines

**Table S1** Overview of full and reduced ANOVA models evaluating unique OTUs in DGRP flies under control and restricted dietary conditions. Diet is treated as a fixed effect, while DGRP line is modelled as a random effect

| Condition | Source | DF | Mean Sq | F Value | p-value |
| --- | --- | --- | --- | --- | --- |
| Control diet | L | 1 | 3.90 | 4.68 | 0.0315 |
|  | $\epsilon$ | 241 | 0.83 | - | - |
| Restricted diet | L | 1 | 8.16 | 7.90 | 0.0054 |
|  | $\epsilon$ | 231 | 1.03 | - | - |
| Both diets | L | 1 | 11.67 | 12.54 | 0.0004 |
|  | D | 1 | 23.33 | 25.07 | <0.0001 |
|  | L×D | 1 | 0.39 | 0.420 | 0.5172 |
|  | $\epsilon$ | 472 | 0.93 | - | - |

**Table S2** Overview of full and reduced ANOVA models evaluating Simpson Index in DGRP flies under control and restricted dietary conditions. Diet is treated as a fixed effect, while DGRP line is modelled as a random effect

| Condition | Source | DF | Mean Sq | F Value | p-value |
| --- | --- | --- | --- | --- | --- |
| Control diet | L | 1 | 1.71 | 1.96 | 0.1629 |
|  | $\epsilon$ | 241 | 0.87 | - | - |
| Restricted diet | L | 1 | 2.45 | 2.20 | 0.1394 |
|  | $\epsilon$ | 231 | 1.11 | - | - |
| Both diets | L | 1 | 0.03 | 0.03 | 0.8552 |
|  | D | 1 | 2.67 | 2.69 | 0.1015 |
|  | L×D | 1 | 4.13 | 4.16 | 0.0418 |
|  | $\epsilon$ | 472 | 0.99 | - | - |

**Table S3** Overview of full and reduced ANOVA models evaluating Shannon Index in DGRP flies under control and restricted dietary conditions. Diet is treated as a fixed effect, while DGRP line is modelled as a random effect

| Condition | Source | DF | Mean Sq | F Value | p-value |
| --- | --- | --- | --- | --- | --- |
| Control diet | L | 1 | 2.68 | 3.05 | 0.0821 |
|  | $\epsilon$ | 241 | 0.88 | - | - |
| Restricted diet | L | 1 | 2.23 | 2.11 | 0.1479 |
|  | $\epsilon$ | 231 | 1.06 | - | - |
| Both diets | L | 1 | 0.01 | 0.01 | 0.9175 |
|  | D | 1 | 13.11 | 13.55 | 0.0003 |
|  | L×D | 1 | 4.90 | 5.07 | 0.0248 |
|  | $\epsilon$ | 472 | 0.97 | - | - |
